# Editorial: Stem cells and kidney regeneration: transforming renal medicine

**DOI:** 10.3389/fbioe.2025.1749288

**Published:** 2025-11-28

**Authors:** Stefano Da Sacco, Benedetta Bussolati, Laura Perin

**Affiliations:** 1 The GOFARR Laboratory, The Saban Research Institute, Division of Urology, Children’s Hospital Los Angeles, Los Angeles, CA, United States; 2 Department of Urology, Keck School of Medicine, University of Southern California, Los Angeles, CA, United States; 3 Department of Medical Sciences, University of Turin, Turin, Italy

**Keywords:** renal regeneration, stem cells, development, stem cells bioproducts, renal disease

The field of renal medicine stands at a critical turning point. With chronic kidney disease (CKD) affecting hundreds of millions worldwide and the limitations of dialysis and transplantation becoming increasingly evident, the need for novel solutions has never been more urgent (GBD, 2023 Chronic Kidney Disease Collaborators, 2025).

Stem cells and stem cell-bioproducts based research is emerging as a potential and versatile approach to address the structural and functional decline of kidney disease, as well as tools to understand the mechanisms regulating development and disease progression (Salybekov et al., 2024; Chang et al., 2025; Kim et al., 2019; Tsuji et al., 2022). The contributions assembled in this Research Topic reflect the innovation in this space, offering not only therapeutic hope but also new conceptual frameworks for understanding kidney regeneration and disease mechanisms.

The emerging idea that the adult kidney may regain regenerative abilities through transcriptional reprogramming is at the base of the study by Pode-Shakked et al.. This study explores the reactivation of developmental genes, SIX2 and OSR1, in adult human kidney epithelial cells, demonstrating how developmental biology can be exploited to induce regenerative processes. By reintroducing SIX2, the Authors observed an enhancement in cellular self-renewal and in the ability to form organized tubular structures *in vivo*, suggesting that adult kidney cells retain plasticity that can be unlocked when cells are stimulated by specific signaling. Yet, the study also serves as a cautionary tale: OSR1, while activating developmental programs, was associated with malignant transformation in a subset of cells, mimicking features of Wilms tumor. This duality of regeneration versus tumorigenesis underscores the fragile balance that must be maintained when manipulating developmental pathways in adult tissues.

Complementary, the work by Gyarmati et al. shifts attention to an often-overlooked player in renal physiology: the macula densa. Traditionally viewed as a sensor of tubular sodium concentration, the macula densa is revealed here as a potent source of regenerative signals. The identification of CCN1, an angiogenic and anti-fibrotic factor, positions the macula densa as a novel therapeutic target and a potential cellular source for regenerative interventions. This discovery frames the macula densa cells as a regenerative niche, underscoring their relevance in the field by leveraging the kidney’s intrinsic repair mechanisms rather than relying solely on exogenous cell replacement.

While cellular therapies hold great promise, the challenges of immunogenicity, scalability, and safety have prompted interest in cell-free alternatives. The review by Ceccotti et al. offers a comprehensive and timely synthesis of the therapeutic potential of mesenchymal stem cell-derived extracellular vesicles (MSC-EVs). These vesicles, enriched with specific regenerative cargo, have demonstrated the ability to modulate inflammation, fibrosis, and oxidative stress. The EV-based approach is particularly attractive due to its versatility and safety profile. By combining the therapeutic effects of stem cells into a nano-sized, acellular format, MSC-EVs circumvent many of the regulatory hurdles associated with live cell therapies. The review not only consolidates the preclinical evidence but also points to early clinical data suggesting feasibility and safety, paving the way for broader EV translational efforts for kidney disease.

Finally, the study by Vax et al. brings the conversation full circle by applying stem cell insights to oncology. Their development of a monoclonal antibody targeting Frizzled 7 (FZD7), a Wnt receptor enriched in Wilms tumor cancer stem cells, exemplifies how regenerative biology can inform cancer therapeutics. By selectively disrupting Wnt signaling in these cells, the antibody effectively reduced tumor growth, a clear evidence of the potential of precision targeting in renal regeneration. This work highlights the potential of stem cell markers not only as tools for regeneration but also as vulnerabilities in cancer, offering a dual benefit in both tissue repair and disease suppression.

Taken together, these studies illustrate a lively and rapidly evolving landscape in renal regenerative medicine that is not limited to the classical view of stem cell therapeutics. By challenging the notions about the irreversibility of kidney damage, they open new avenues for clinically relevant interventions. At the same time, they also remind us of the complexities related to reactivating developmental programs in adult tissues. The path forward to precision medicine of the kidney will require not only scientific accountability but also careful safety considerations.

Looking ahead, several hurdles will need to be addressed. First, the field must continue to refine strategies that balance efficacy with safety, particularly when manipulating potent developmental genes. Second, there is a need for more physiologically relevant models that can bridge the gap between preclinical promise and clinical application. Third, standardization of cell sources, EV isolation protocols, and delivery methods will be critical for regulatory approval and widespread adoption. Finally, the integration of personalized medicine approaches, including patient-derived cells and organoids, will be essential to tailor therapies to individual disease profiles and genetic backgrounds.

In sum, the contributions in this Research Topic, while presenting recent discoveries related to kidney regeneration, redefine its possibilities. These studies offer us a glimpse into a future where kidney failure will be a treatable condition, where regeneration replaces replacement, and where the kidney’s own biology becomes its best medicine.

## References

[B1] Chang S. Wang Y. Wu C. Chen X. (2025). Research trends and hotspots of kidney disease and stem cell therapy from 2009 to 2025: a bibliometric analysis. Int. Urology Nephrol. 10.1007/s11255-025-04732-7 40839058

[B2] GBD 2023 Chronic Kidney Disease Collaborators (2025). Global, regional, and national burden of chronic kidney disease in adults, 1990-2023, and its attributable risk factors: a systematic analysis for the global burden of disease study 2023. Lancet, 7, (25) S0140–6736. 10.1016/S0140-6736(25)01853-7 41213283

[B3] Kim M. W. Ko I. K. Atala A. Yoo J. J. (2019). Cell-derived secretome for the treatment of renal disease. Child. Kidney Dis. 23, 67–76. 10.3339/jkspn.2019.23.2.67

[B4] Salybekov A. A. Kinzhebay A. Kobayashi S. (2024). Cell therapy in kidney diseases: advancing treatments for renal regeneration. Front. Cell Dev. Biol. 12, 1505601. 10.3389/fcell.2024.1505601 39723242 PMC11669058

[B5] Tsuji K. Kitamura S. Wada J. (2022). Potential strategies for kidney regeneration with stem cells: an overview. Front. Cell Dev. Biol. 10, 892356. 10.3389/fcell.2022.892356 35586342 PMC9108336

